# Chronic Ethanol Differentially Modulates Glutamate Release from Dorsal and Ventral Prefrontal Cortical Inputs onto Rat Basolateral Amygdala Principal Neurons

**DOI:** 10.1523/ENEURO.0132-19.2019

**Published:** 2020-03-04

**Authors:** Molly M. McGinnis, Brian C. Parrish, Ann M. Chappell, Nancy J. Alexander, Brian A. McCool

**Affiliations:** Department of Physiology and Pharmacology, Wake Forest School of Medicine, Winston-Salem, North Carolina 27157

**Keywords:** infralimbic, patch-clamp electrophysiology, prelimbic, presynaptic

## Abstract

The medial prefrontal cortex (mPFC) and the basolateral amygdala (BLA) have strong reciprocal connectivity. Projections from the BLA to the mPFC can drive innate, anxiety-related behaviors, but it is unclear whether reciprocal projections from the mPFC to BLA have similar roles. Here, we use optogenetics and chemogenetics to characterize the neurophysiological and behavioral alterations produced by chronic ethanol exposure and withdrawal on dorsal mPFC (dmPFC) and ventral mPFC (vmPFC) medial prefrontal cortical terminals in the BLA. We exposed adult male Sprague Dawley rats to chronic intermittent ethanol (CIE) using vapor chambers, measured anxiety-like behavior on the elevated zero maze, and used electrophysiology to record glutamatergic and GABAergic responses in BLA principal neurons. We found that withdrawal from a 7 d CIE exposure produced opposing effects at dmPFC (increased glutamate release) and vmPFC (decreased glutamate release) terminals in the BLA. Chemogenetic inhibition of dmPFC terminals in the BLA attenuated the increased anxiety-like behavior we observed during withdrawal. These data demonstrate that chronic ethanol exposure and withdrawal strengthen the synaptic connections between the dmPFC and BLA but weakens the vmPFC–BLA pathway. Moreover, facilitation of the dmPFC–BLA pathway during withdrawal contributes to anxiety-like behavior. Given the opposing roles of dmPFC–BLA and vmPFC–BLA pathways in fear conditioning, our results suggest that chronic ethanol exposure simultaneously facilitates circuits involved in the acquisition of and diminishes circuits involved with the extinction of withdrawal-related aversive behaviors.

## Significance Statement

Accumulating evidence suggests that the medial prefrontal cortex (mPFC) and its projections to the basolateral amygdala (BLA) bidirectionally modulate fear-related behaviors. Since the neuronal circuits for fear and anxiety are thought to overlap, we sought to examine the role of dorsal and ventral subdivisions of the mPFC and their inputs to the BLA in regulating anxiety. Specifically, we focused on alcohol withdrawal-induced anxiety-like behavior, which is a commonly reported cause of relapse in humans with alcoholism. In our study, we used optogenetics and chemogenetics to demonstrate, for the first time, that withdrawal from chronic ethanol exposure strengthens dorsal mPFC (dmPFC) synapses, but weakens ventral mPFC synapses in the BLA and that inhibiting glutamate release from dmPFC terminals in the BLA reduces anxiety-like behavior.

## Introduction

Decades of epidemiological and clinical studies have highlighted the relationship between alcohol use disorder (AUD) and anxiety disorders (Smith and Randall, 2012). Comorbidity between these disorders is highly prevalent and is associated with a complex clinical presentation that makes diagnosis and treatment challenging. Anxiety disorders in individuals with AUD are largely thought to be a lingering consequence of alcohol withdrawal (WD) and can contribute to both the maintenance of pathologic alcohol use and relapse (Schuckit and Hesselbrock, 1994; Kushner et al., 2000). The standard pharmacotherapy and psychotherapy protocols for AUD and comorbid anxiety have had limited success, with ∼80% of individuals relapsing following treatment (Driessen, 2001; Kushner et al., 2005).

The identification of emotional brain circuits that are vulnerable to alcohol dependence is a crucial step for the development of more effective AUD treatments. In this context, the basolateral amygdala (BLA) is an integral component within the neural circuitry regulating fear and anxiety (Davis, 1992; Gallagher and Chiba, 1996; Tovote et al., 2015). Glutamatergic pyramidal neurons within the BLA comprise ∼85% of the cell population and are the primary projection neurons (McDonald, 1982, 1992). These cells receive glutamatergic inputs from a variety of both cortical and subcortical regions, which convey sensory-, executive-, and memory-related information (Ottersen, 1982; Cassell and Wright, 1986; van Vulpen and Verwer, 1989). In turn, these pyramidal neurons project to a variety of downstream brain regions to influence both cognitive and physiologic responses to emotionally relevant stimuli. The remaining cell population consists of GABAergic interneurons, which exert profound inhibitory control over the pyramidal neurons (Carlsen, 1988; Muller et al., 2006). The generation of emotional responses is predominantly determined by BLA principal neuron activity and, thus, the balance between excitatory and inhibitory neurotransmission onto these cells.

The prefrontal cortex provides powerful top–down executive control over limbic brain regions like the amygdala to regulate the expression of affective states (Phelps and LeDoux, 2005). In support of this, the dorsal and ventral subdivisions of the medial prefrontal cortex (mPFC) share robust reciprocal connectivity with principal neurons in the BLA (Little and Carter, 2013; McGarry and Carter, 2017), and these circuits have been implicated in controlling a variety of emotion-related behaviors (Sotres-Bayon and Quirk, 2010; Arruda-Carvalho and Clem, 2015). Importantly, these distinct mPFC subregions and their BLA projections appear to play dichotomous roles in learned fear behaviors with dorsal mPFC [dmPFC; e.g., prelimbic cortex (PL)] facilitating the acquisition/expression-conditioned responses and the ventral PFC [vmPFC; e.g., infralimbic cortex (IL)] facilitating the extinction of these learned associations (Sierra-Mercado et al., 2011; Arruda-Carvalho and Clem, 2014; Senn et al., 2014; Bukalo et al., 2015; Bloodgood et al., 2018). Together, these data provide strong evidence that the dmPFC and vmPFC projections to the BLA are important pathways for the regulation of fear. However, the extent to which these circuits might contribute to pathologic conditions, such as during alcohol dependence, remains to be fully elucidated.

Evidence suggests that the cellular and molecular mechanisms underlying fear and anxiety are similar in both animals and humans and that these processes are likely mediated by overlapping neuronal circuits. Therefore, we hypothesized that projections from the dmPFC and vmPFC to the BLA would be vulnerable to chronic ethanol exposure and may regulate the behavioral consequences of alcohol dependence. A commonly used method for inducing ethanol dependence in animal models is chronic intermittent ethanol (CIE) exposure. Using this model, the literature indicates that withdrawal from CIE increases innate anxiety-like behaviors (Roberto et al., 2008; Morales et al., 2015) and markedly increases glutamatergic synaptic activity recorded from BLA pyramidal neurons (Christian et al., 2012, 2013; Morales et al., 2018). These data suggest that glutamate inputs, from regions such as the mPFC, are potentially altered by chronic ethanol exposure and withdrawal. Notably, CIE impairs fear extinction and remodels glutamatergic mPFC neurons (Holmes et al., 2012) providing further evidence that specific mPFC projections to the BLA may be altered by chronic ethanol exposure and mediate withdrawal-induced anxiety. Here, we took a circuit-based approach using electrophysiology in combination with optogenetics and chemogenetics to examine dmPFC and vmPFC terminals in the BLA, the effects of CIE exposure, and their role in regulating anxiety-like behavior during withdrawal.

## Materials and Methods

### Animals

Male Sprague Dawley rats were obtained from Envigo. On arrival, rats were pair housed, given food and water *ad libitum*, and maintained on a reverse 12 h light/dark cycle (lights on at 9:00 P.M.). Rats were ∼5 weeks of age at the time of virus microinjection and ∼10 weeks of age at the time of behavioral manipulations/electrophysiology recordings. All animal care procedures were in accordance with the National Institutes of Health *Guide for the Care and Use of Laboratory Animals* and experimental procedures were approved in advance by the Institutional Animal Care and Use Committee at Wake Forest University School of Medicine.

### Stereotaxic surgery

#### Viral microinjection

Rats (*n* = 115; weight, ∼100 g) were induced using 3–5% isoflurane anesthesia and maintained under continuous 1–3% isoflurane for the duration of the procedure with the oxygen flowmeter set to 1.0 L/min. Bilateral microinjections (1 μl/side) of adeno-associated viral vectors containing channelrhodopsin 2 [ChR2; AAV5-CamKIIα-hChR2(H134R)-EYFP (enhanced yellow fluorescent protein); UNC Vector Core, University of North Carolina, Chapel Hill, Chapel Hill, NC], G_i_-coupled designer receptor exclusively activated by designer drugs [DREADD; AAV5-CamKIIα-hM4D(G_i_)-mCherry; Addgene], G_q_-coupled DREADD [AAV5-CamKIIα-hM3D(G_q_)-mCherry; Addgene], or a control fluorophore (AAV5-CamKIIα-mCherry; UNC Vector Core) were delivered at a rate of 0.1 μl/min over 10 min (1 μl total volume) using a syringe pump (Harvard Apparatus). Injectors were left in place for an additional 5 min to allow for virus to diffuse. The medial prefrontal cortex was targeted using an automated stereotaxic system (StereoDrive, Neurostar) with the following coordinates (in mm relative to bregma): dmPFC: anteroposterior (AP), 3.20 ± 0.50; dorsoventral (DV), 2.75); or vmPFC: AP, 3.20 ± 0.50 ml; DV, 4.75. Targeting the upper portion of the dmPFC and the lower portion of the vmPFC allowed for maximal spatial segregation between subregions. At the end of surgery, rats were given 3 mg/kg ketoprofen (Patterson Veterinary Supply) for pain management and 2 ml of warmed sterile saline. Sutures were removed 1 week later, and animals were pair housed. Rats were allowed a total of 4 weeks to recover and for virus to express before further experimentation. Injection sites were confirmed by collecting coronal slices of the mPFC and visualizing EYFP and mCherry using fluorescence microscopy postmortem. Rats with unintended viral placement were excluded from the study.

#### Cannulation

For the behavioral studies using BLA microinjections, animals (*n* = 48; weight, ∼300 g) underwent a second surgery 4 weeks after virus injection to implant bilateral guide cannulae. Rats were deeply anesthetized with pentobarbital sodium (50 mg/kg, i.p.), and 15-mm-long, stainless steel guide cannulae (26 gauge; Plastics One) were implanted using a stereotaxic instrument, terminating 1 mm above the BLA using the following coordinates (in mm from bregma): AP, −2.80 ± 5.00; DV, 7.80. Dental cement (Lang Dental Manufacturing) was affixed and stabilized the guide cannulae to the skull. Obturators (15 mm long; Plastics One) maintained the patency of the guide cannulae. During a 5 d recovery period, rats were extensively handled and repeatedly exposed to the manipulations associated with the microinjection procedure. Correct placement of the guide cannulae was confirmed postmortem by preparing coronal slices from fixed tissue.

### Chronic intermittent ethanol vapor exposure

Pair-housed rats were exposed to CIE vapor for 3 or 7 consecutive days, using standard procedures (Läck et al., 2007; Robinson et al., 2016). Briefly, animals in their home cages were placed inside larger, custom-built Plexiglas chambers (Triad Plastics). At the beginning of the light cycle (lights on at 9:00 P.M.), ethanol vapor was pumped into the chambers at a constant rate (16 L/min) and maintained at ∼25 mg/L throughout the exposure for 12 h/d. Air-exposed (AIR) control animals were similarly housed, except they received room air only. Animals were weighed daily, and tail blood samples were collected twice during the CIE exposure (day 2 and day 5) in a subset of animals to monitor blood ethanol concentrations (BECs) and adjust ethanol vapor levels as necessary. Average BECs during the CIE exposure were 241.29 ± 12.97 mg/dl, as determined by a standard, commercially available alcohol dehydrogenase/NADH (nicotinamide adenine dinucleotide plus hydrogen) enzymatic assay (Carolina Liquid Chemistries). These BECs are in the standard range (150–275 mg/dl) to produce dependence. All behavioral assays and electrophysiology recordings were conducted 24 h after the last ethanol (or air) exposure.

### Elevated zero maze

Twenty-four hours after the last ethanol (or air) exposure, rats were microinjected with either artificial CSF (aCSF) or 300 μm clozapine-*N*-oxide (CNO; Tocris Bioscience) and then tested on the elevated zero maze (EZM; Med Associates) to assess anxiety-like behavior. The EZM is a circular maze consisting of two elevated closed or open sections, without the issue of an ambiguous center position found in the elevated plus maze. For microinjections, obturators were removed and 16-mm-long, 33 gauge injection cannulae were inserted into the guide cannulae such that their tips extended 1 mm below the ventral aspect of the guide cannulae. Drug solutions were bilaterally infused into the BLA over a 1 min period with 0.5 μl total volume delivered per side. Injectors were left in place for an additional minute before being removed. Animals were placed on the EZM illuminated by a 7.5 W incandescent bulb 5 min after the microinjection (Nobre, 2016). An ace monochrome camera (Basler) along with EthoVision XT Video-Tracking Software (Noldus) were used to track center point, tail base, and nose point throughout the 5 min test to assess anxiety-like behavior and general locomotion. Between animals, the apparatus was cleaned with warm water and mild soap, and then thoroughly dried. The percentage of time was calculated by dividing the time (in seconds) spent collectively in both open areas by the entire time of the test, which was 300 s.

### Electrophysiology

#### Slice preparation

Animals were anesthetized with isoflurane and then decapitated using a guillotine. Brains were rapidly removed and incubated for 5 min in an ice-cold sucrose-modified aCSF solution containing the following (in mm): 180 sucrose, 30 NaCl, 4.5 KCl, 1 MgCl_2_ · 6H_2_O, 26 NaHCO_3_, 1.2 NaH_2_PO_4_, 10 d-glucose, and 0.10 ketamine, equilibrated with 95% O_2_ and 5% CO_2_. Coronal slices (400 μm) containing the BLA were collected using a VT1200/S Vibrating Blade Microtome (Leica), incubated for ≥1 h at room temperature (∼25°C) in oxygenated in a standard aCSF solution containing the following (in mm): 126 NaCl, 3 KCl, 1.25 NaH_2_PO_4_, 2 MgSO_4_ · 7 H_2_O, 26 NaHCO_3_, 10 d-glucose, and 2 CaCl_2_ · 2H_2_O. For low calcium recordings, an altered aCSF solution with 3 mm MgSO_4_ and 1 mm CaCl_2_ were used to keep extracellular divalent cation concentrations constant across conditions. All chemicals were obtained from Sigma-Aldrich or Tocris Bioscience.

#### Whole-cell patch-clamp recording

BLA slices were transferred to a submersion-type recording chamber that was continuously perfused at a rate of 2 ml/min with oxygenated, room temperature (∼25°C) aCSF. Synaptic responses were recorded using electrodes filled with an intracellular solution containing the following (in mm): 145 CsOH, 10 EGTA, 5 NaCl, 1 MgCl_2_ · 6 H_2_O, 10 HEPES, 4 Mg-ATP, 0.4 Na-GTP, 0.4 QX314, and 1 CaCl_2_·2H_2_O, with pH adjusted to ∼7.3 with gluconic acid, and osmolarity adjusted to ∼285 Osm/L with sucrose. Optogenetic (ChR2) responses were evoked using a 473 nm laser connected to a fiber optic cable (Thorlabs); the naked end of the cable was placed just above the stria terminalis on the medial side of the BLA. Five millisecond laser pulses were used to activate channelrhodopsin found in the mPFC terminals. Sweeps were recorded every 30 s, and light stimulation intensities were submaximal and normalized to elicit synaptic responses with amplitudes of ∼100 pA. Glutamatergic synaptic currents were recorded at a membrane holding potential of −70 mV and pharmacologically isolated using the GABA_A_ antagonist picrotoxin (100 μm). For measures of EPSC/IPSC (E/I) ratios, picrotoxin was omitted from the extracellular solution. In some recordings, 1 μm tetrodotoxin (TTX; Tocris Bioscience) and 20 mm 4-aminopyridine (4-AP; Tocris Bioscience) were included to isolate monosynaptic responses (Petreanu et al., 2009; Cruikshank et al., 2010). Data were acquired at 5 kHz and low-pass filtered at 2 kHz via an Axopatch 700B Amplifier and pClamp 10 software (Molecular Devices). Presumptive principal neurons were included based on their electrophysiological characteristics of high membrane capacitance (>100 pF) and low access resistance (≤25 MΩ; Washburn and Moises, 1992). Cells that did not meet these criteria or cells in which access resistance, capacitance, or holding current changed ≥20% during the recording were excluded from analysis.

#### Paired-pulse ratio

Two 5 ms light stimuli of equal intensity were delivered to the stria terminalis at an interstimulus interval of 50 ms, with relative amplitudes produced at this short interval being traditionally viewed as an indicator of presynaptic release probability (Fioravante et al., 2011). The paired-pulse ratio (PPR) was conservatively calculated using the evoked EPSC amplitudes as follows: [Peak 2 amplitude − Peak 1 amplitude]/Peak 1 amplitude. The average paired-pulse ratio was determined for a 5 min baseline and was compared with the average ratio of the final 5 min of drug application and washout.

### Western blotting

Total protein was prepared from GFP-positive regions of ChR2-injected mPFC from air-exposed and CIE-exposed animals (*N* = 16). Ten micrograms of total protein was fractioned on 4–20% 18-well Criterion TGX Precast Midi Protein Gels (catalog #567-8094, Bio-Rad) and transferred to a nitrocellulose membrane with a Bio-Rad Trans Blot Turbo Transfer System and preassembled membrane stacks (catalog #1704270, Bio-Rad). Total lane protein transfer was detected using a Pierce Reversible Protein Stain Kit (catalog #PI24580, Thermo Fisher Scientific). GFP was targeted with chicken anti-GFP primary antibody (1 μg/ml; catalog #GFP-1020, Aves; RRID:AB_10000240), which was detected with peroxidase-labeled goat anti-chicken secondary antibody (1:5000; catalog #H-1004, Aves; RRID:AB_2313517) and SuperSignal West Dura Extended Duration Substrate Enhanced Chemiluminescence (catalog #34076, Thermo Fisher Scientific). Immunoreactive band intensity was quantified from digital images captured on a charge-coupled device camera and normalized to total lane protein using a Bio-Rad Chemi-Doc XRS Imaging System, and Image Lab Analysis software (Bio-Rad).

### Experimental design and statistical analysis

Primary statistical analyses were conducted using Prism 5 (GraphPad Software). Data were analyzed using ANOVA or *t* tests depending on the experimental design, with Bonferroni *post hoc* tests used to determine the locus of effect as appropriate. A value of *p* < 0.05 was considered to be statistically significant. All data are presented as the mean ± SEM throughout the text and figures.

## Results

### Neurons from the dmPFC and vmPFC make monosynaptic glutamatergic synapses with principal neurons in the BLA

**Figure 1.**
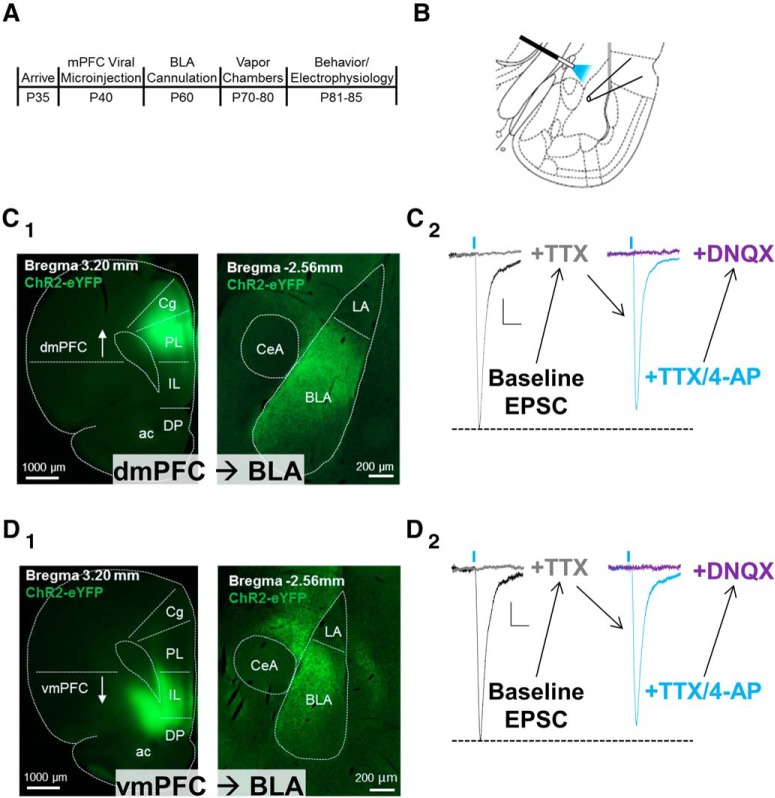
The dorsal and ventral subdivisions of the mPFC make dense, monosynaptic glutamatergic synaptic connections with BLA principal neurons. ***A***, Experimental timeline with procedures on the top and approximate age of the rats in postnatal days on the bottom. ***B***, Schematic depicting the typical placement of the optical “stimulator” and patch electrode for electrophysiology recordings. The optical fiber delivering 470 nm blue light was placed just medial of the BLA above the stria terminalis to activate the channelrhodopsin-expressing mPFC inputs. BLA principal neurons were patched along the dorsomedial aspect of the subdivision where the YFP-expressing terminals were most dense. ***C_1_***, ***D_1_***, Representative fluorescent images of the mPFC injection site (left) and the resulting terminals in the BLA (right) 4 weeks after the injection of channelrhodopsin into the dmPFC (***C_1_***) or vmPFC (***D_1_***), respectively. ***C_2_***, ***D_2_***, Representative traces of light-evoked oEPSCs recorded from dmPFC–BLA (***C_2_***) or vmPFC–BLA (***D_2_***) synapses, respectively, in the presence of various agents. See text for details. Calibration: 20 ms, 20 pA. Blue vertical dashes represent the approximate onset of optogenetic stimulation (5 ms duration). ac, Anterior commissure; LA, lateral amygdala.

Injection of channelrhodopsin-YFP targeting either the dmPFC (Fig. 1*C1*) or vmPFC (Fig. 1*D1*) results in robust YFP fluorescence along the stria terminalis, just dorsal of the central nucleus of the amygdala (CeA), and within the BLA. There were little to no YFP-expressing terminals in the lateral amygdala (LA) or the CeA, which is a typical innervation pattern for projections from the rat mPFC (Reppucci and Petrovich, 2016; McGarry and Carter, 2017). Patterns of fluorescence from dmPFC and vmPFC injections were qualitatively similar across the anterior–posterior axis of the BLA and appeared largely localized to the anterior subdivision.

To demonstrate monosynaptic projections from mPFC to BLA principal neurons, we recorded light-evoked EPSCs [i.e., optical EPSCs (oEPSCs)] in the presence of TTX (sodium channel blocker) and 4-AP (potassium channel blocker; Fig. 1*C2*,*D2*). One micromolar TTX completely inhibited the oEPSCs; and, application of 1 mm 4-AP in the presence of TTX largely reversed this inhibition. The amplitudes of EPSCs collected in the presence of TTX and 4-AP were 89.5 ± 22.3% (dmPFC) and 81.4 ± 11.6% (vmPFC) of the baseline EPSC amplitudes, suggesting that a majority of the baseline synaptic responses were monosynaptic, similar to previous reports (Cho et al., 2013; Little and Carter, 2013). The AMPA receptor antagonist DNQX (20 μm), abolished the monosynaptic EPSC and confirmed that these oEPSCs were glutamatergic.

### Withdrawal from 3 d of CIE does not alter glutamate release from mPFC terminals in the BLA

**Figure 2.**
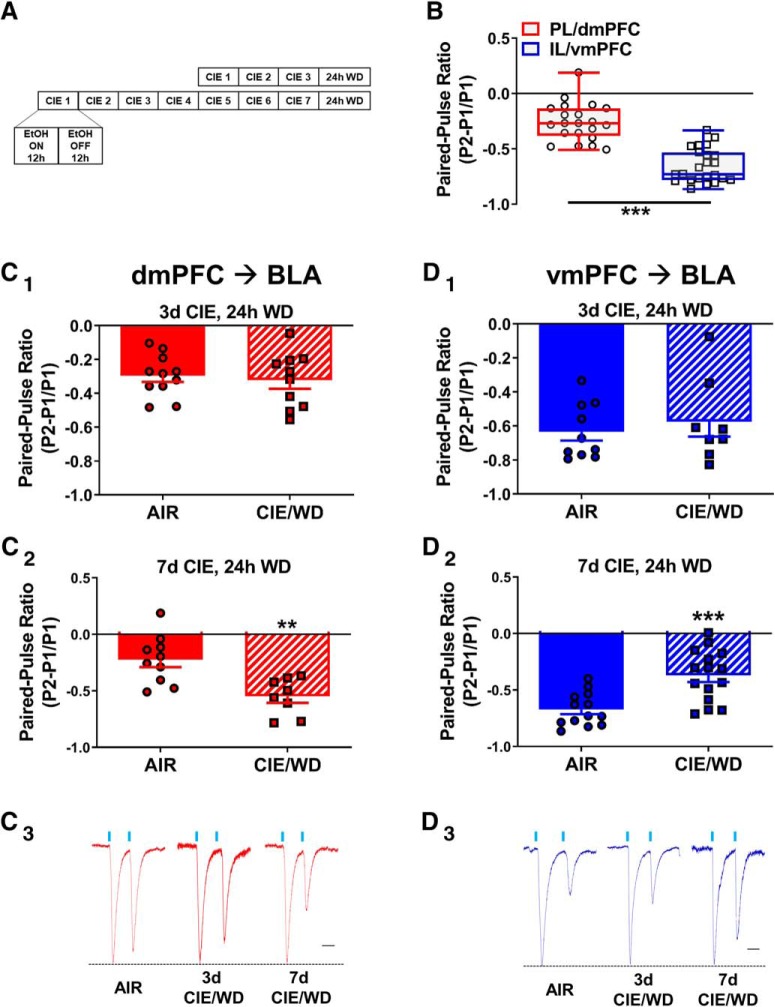
WD from 7 d, but not 3 d, of CIE bidirectionally modulates glutamate release from dmPFC and vmPFC terminals in the BLA. ***A***, Timeline demonstrating the 3 and 7 d CIE exposures. Each day, rats were exposed to 12 h of ethanol (EtOH) and 12 h of air. All electrophysiology recordings were conducted 24 h after the last exposure. Control animals were identically housed but only exposed to air. ***B***, Optogenetically evoked glutamate PPRs recorded from BLA principal neurons in air-exposed controls were significantly different between dmPFC (*N* = 21, circles) and vmPFC (*N* = 23, squares) terminals. For the “box and whisker” plots, the box encompasses the 25th to 75th percentiles of the population, and the line inside the box represents the median; the whiskers represent minimum and maximum values. ***C_1_***, No effect of 3 d CIE on the PPR recorded from dmPFC–BLA neurons in AIR (*N* = 11) vs CIE/WD (*N* = 10) animals. Bars represent the mean ± SEM, and individual neurons are indicated by circles or squares for this panel and the remaining figures. ***C_2_***, Significant decrease in the PPR after 7 d of CIE in dmPFC–BLA neurons, AIR (*N* = 10) vs CIE/WD (*N* = 8) indicates increased glutamate release probability. ***D_1_***, No effect of 3 d CIE on PPR recorded from vmPFC–BLA neurons in AIR (*N* = 10) vs CIE/WD (*N* = 8) animals. ***D_2_***, A significant increase in the PPR after 7 d of CIE in vmPFC–BLA neurons, AIR (*N* = 13) vs CIE/WD (*N* = 15) indicates decreased glutamate release probability. ***C_3_***, ***D_3_***, Representative traces of PPR recorded from dmPFC–BLA (***C_3_***) or vmPFC–BLA (***D_3_***) neurons from AIR (left), 3 d CIE/WD (middle), and 7 d CIE/WD (right). Representative traces were normalized using peak 1. Scale bar, 50 ms. **p < 0.01; ***p < 0.001, unpaired *t* test.

After confirming that we could reliably evoke and record monosynaptic glutamatergic oEPSCs from dmPFC and vmPFC terminals in the BLA, we examined the effects of chronic ethanol exposure and withdrawal on these circuits. We first exposed animals to 3 d of CIE based on recently published data demonstrating that a single 3 d CIE exposure is sufficient to produce presynaptic facilitation at stria terminalis inputs to BLA neurons (Morales et al., 2015). Surprisingly, withdrawal from 3 d of CIE exposure did not significantly alter the glutamate release probability from dmPFC (AIR: −0.30 ± 0.04, *N* = 11; CIE/WD: −0.32 ± 0.05, *N* = 10; unpaired *t* test, AIR vs CIE/WD: *t*_(19)_ = 0.4170, *p* = 0.68) or vmPFC (AIR: −0.63 ± 0.05, *N* = 10; CIE/WD: −0.57 ± 0.09, *N* = 8; unpaired *t* test, AIR vs CIE/WD: *t*_(16)_ = 0.6094, *p* = 0.55) terminals in the BLA (Fig. 2*C1*,*D1*, respectively). Glutamate release probabilities recorded from air-exposed control animals (Fig. 2*B*) were significantly different between dmPFC terminals (PPR = −0.26 ± 0.04, *n* = 21) and vmPFC terminals (PPR = −0.66 ± 0.03, *n* = 23; unpaired *t* test, *t*_(42)_ = 8.090, *p* < 0.001).

### Withdrawal from 7 d of CIE bidirectionally modulates glutamate release from dmPFC and vmPFC terminals in the BLA

Next, we exposed animals to a longer, 7 d CIE exposure. This longer exposure significantly increased the PPR from dmPFC terminals (AIR: −0.22 ± 0.07, *N* = 10; CIE/WD: −0.55 ± 0.06, *N* = 8; unpaired *t* test, AIR vs CIE/WD: *t*_(16)_ = 3.567, *p* < 0.01; Fig. 2*C2*) and significantly decreased in the PPR from vmPFC terminals (AIR: −0.67 ± 0.04, *N* = 13: CIE/WD: −0.37 ± 0.06, *N* = 15; unpaired *t* test, AIR vs CIE/WD: *t*_(26)_ = 4.100, *p* < 0.001; Fig. 2*D2*) compared with air-exposed control animals. As the paired-pulse ratio is inversely related to release probability, these data suggest that chronic ethanol exposure simultaneously facilitates glutamate release from dmPFC-BLA terminals and suppresses glutamate release from vmPFC-BLA terminals. Using dmPFC and vmPFC tissue from a separate cohort of ChR2-injected AIR and 7 d CIE/WD animals (*N* = 16), we examined YFP protein levels using Western blot analysis. A two-way ANOVA of normalized GFP expression across treatment and brain region found no main effects or interactions, as follows: exposure condition (AIR vs CIE/WD: *F*_(1,12)_ = 0.008762, *p* = 0.93), brain region (dmPFC vs vmPFC: *F*_(1,12)_ = 0.0218, *p* = 0.89), and interaction (exposure × region: *F*_(1,12)_ = 0.0218, *p* = 0.89). These data indicate that CIE/WD does not alter ChR2/YFP expression from the viral construct in either the dmPFC or vmPFC.

### Reducing extracellular calcium levels disrupts glutamate release from vmPFC terminals but not dmPFC terminals in the BLA of air-exposed controls

**Figure 3.**
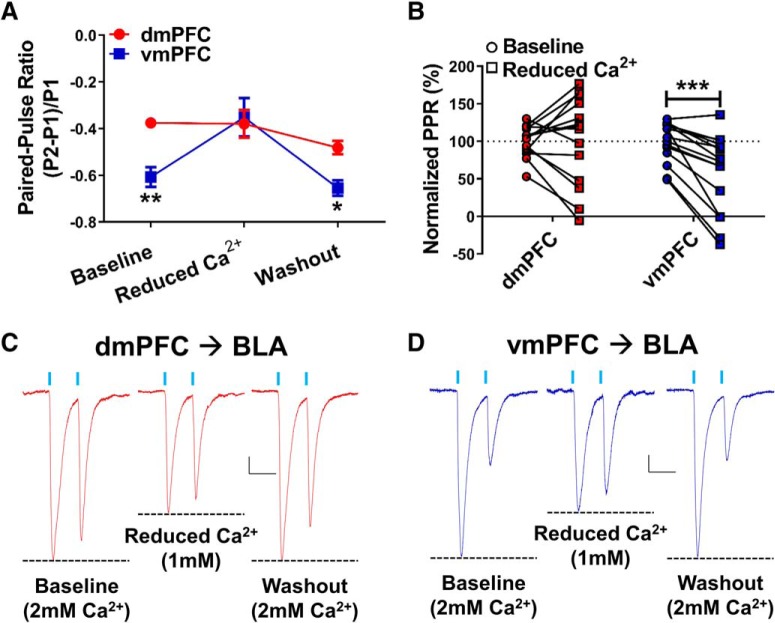
Reducing the extracellular calcium concentration preferentially disrupts glutamate release probability in vmPFC-BLA, but not dmPFC-BLA, terminals in air-exposed control neurons. ***A***, Paired-pulse ratios recorded from dmPFC or vmPFC terminals in the BLA during baseline (2 mm Ca^2+^), reduced extracellular calcium (1 mm Ca^2+^), and washout (2 mm Ca^2+^). Significant differences in the PPR values measured from dmPFC (*N* = 14) and vmPFC (*N* = 15) terminals at baseline and washout but not in the presence of low calcium. Points represent the mean ± SEM for each group. ***B***, Normalized PPR values, here expressed as a percentage of the mean baseline PPR within each input, allow for direct comparisons across inputs of the reduced Ca^2+^ effect on glutamate release probability. Significant differences between baseline and reduced calcium for vmPFC synapses but not dmPFC. *p < 0.05, **p < 0.01, ***p < 0.001, Bonferroni’s post-test results following two-way repeated-measures ANOVA (see text for details). ***C***, ***D***, Representative traces of light-evoked paired-pulse responses with a 50 ms interstimulus interval from dmPFC–BLA (***C***) and vmPFC–BLA (***D***) projections under baseline (left, 2 mm), reduced calcium (middle, 1 mm), and washout (right, 2 mm) conditions. Dashed lines highlight relative differences between first- and second-response oEPSC amplitudes in each condition. Calibration: *x* = 50 ms, *y* = 20 pA.

While examining the effects of chronic ethanol exposure on projections from the dmPFC and vmPFC, we found that the release probabilities at these distinct inputs were significantly different from each other in air-exposed control animals (Fig. 2*B*). It is well established that calcium plays an important role in neurotransmitter release (Neher and Sakaba, 2008). Therefore, we examined the effects of manipulating extracellular calcium concentrations on presynaptic glutamate release at these inputs in ethanol-naive animals. Reducing extracellular calcium levels from 2 to 1 mm reduced first-response amplitudes of paired oEPSCs evoked from both dmPFC and vmPFC inputs (Fig. 3*C*,*D*); and there was no significant difference between inputs with respect to the magnitude of this inhibition (dmPFC: 43.8 ± 4.9%, *n* = 14; vmPFC: 47.2 ± 7.3%, *n* = 15; *t* test, *t* = 0.3844, *p* = 0.704). The 1 mm extracellular calcium also had no effect on glutamate paired-pulse ratios measured from dmPFC terminals (baseline: −0.38 ± 0.02; low calcium: −0.38 ± 0.06; washout: −0.48 ± 0.03; *N* = 14; Fig. 3*A*,*C*). However, the release probability from vmPFC terminals was significantly decreased when calcium was lowered (Fig. 3*A*,*D*: baseline: −0.61 ± 0.04; low calcium: −0.35 ± 0.08; washout: −0.66 ± 0.03, *N* = 15). A two-way repeated-measures ANOVA revealed a main effect of calcium levels (*F*_(2,54)_ = 19.19, *p* < 0.0001), a main effect of input (*F*_(1,54)_ = 4.74, *p* < 0.05), and a significant interaction (*F*_(2,54)_ = 8.55, *p* < 0.001). Bonferroni post-tests (dmPFC vs vmPFC) confirmed observations from the CIE studies showing that there was a significant difference between the release probabilities from dmPFC and vmPFC terminals at baseline (*t* = 3.318, *p* < 0.01) and during washout (*t* = 2.470, *p* < 0.05) when the calcium levels were restored (Fig. 3*A*). Interestingly, lowering calcium levels “normalized” vmPFC and dmPFC release probability to the point where they were no longer significantly different (*t* = 0.4048, *p* > 0.05). Normalized PPR (Fig. 3*B*) data were expressed as a percentage of the average baseline PPR within each projection to emphasize the effects of the calcium manipulation. Two-way repeated-measures ANOVA of these data revealed a main effect of calcium level (*F*_(1,27)_ = 7.357, *p* < 0.05) and a significant interaction (*F*_(1,27)_ = 8.227, *p* < 0.01). Planned Bonferroni post-tests (baseline vs reduced calcium) revealed a significant difference in the effect of calcium levels on vmPFC inputs (*t* = 4.016, *p* < 0.001) but not dmPFC inputs (*t* = 0.1084, *p* > 0.05). Thus, lowering extracellular calcium concentrations from 2 to 1 mm reduced glutamate release from vmPFC terminals by 42.2 ± 13.5% relative to baseline, but did not affect release from dmPFC terminals in the BLA (101.2 ± 15.8%; Fig. 3*B*). These data suggest that the baseline differences in release probability between dmPFC and vmPFC inputs, and potentially their differential sensitivity to chronic ethanol exposure, are likely related to calcium-dependent processes within these terminals.

### Bath application of CNO decreases glutamate release from G_i_-DREADD expressing dmPFC terminals in the BLA

**Figure 4.**
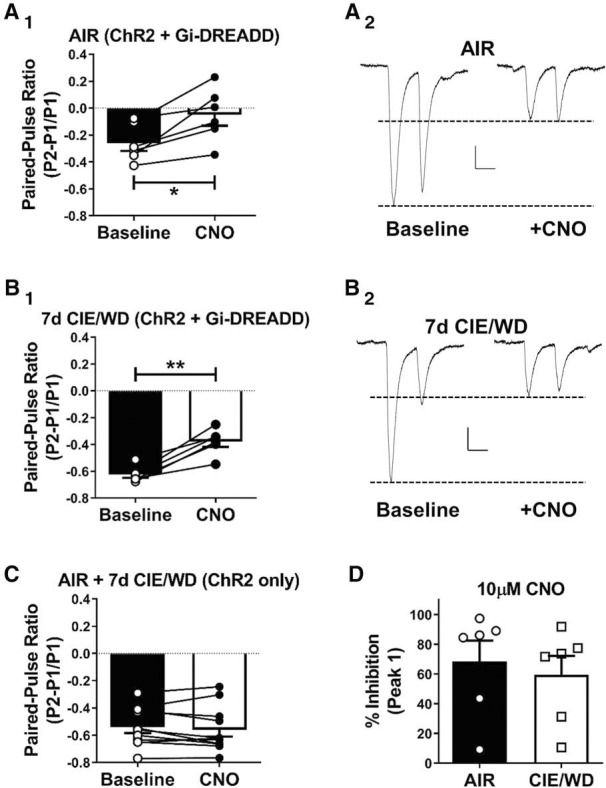
Bath application of CNO decreases glutamate release from dmPFC-BLA terminals expressing the hM4Di-DREADD receptor. ***A_1_***, ***B_1_***, Light-evoked paired-pulse ratios recorded from dmPFC-BLA terminals expressing ChR2 and G_i_-coupled DREADDs at baseline (closed bars, open circles) and during the bath application of CNO (10 μm; open bars, closed circles) in air-exposed control neurons (*N* = 6; ***A_1_***) and in 7 d CIE/WD neurons (*N* = 6; ***B_1_***). Note that some individual data points are superimposed due to the scale. CNO significantly decreased the oEPSC amplitude and increased the PPR in all of the cells. *p < 0.05; **p < 0.01; paired *t* test. ***A_2_***, ***B_2_***, Representative traces of paired-pulse responses recorded from AIR (***A_2_***) or CIE/WD (***B_2_***) neurons at baseline (left) and in the presence on CNO (right). Calibration: *x* = 50 ms, *y* = 20 pA. ***C***, PPRs recorded from AIR and CIE/WD BLA neurons (*N* = 11) with dmPFC-BLA terminals expressing only ChR2 (no DREADD controls). CNO did not significantly change glutamate the release probability from terminals without expressing the DREADD construct. ***D***, The percentage of inhibition by CNO, calculated from the first response amplitude (***A_2_***, ***B_2_***), was not different between AIR and CIE/WD recordings.

Since increased glutamate release in the BLA can drive principal neuron activation and contribute to increases in anxiety-like behavior, we hypothesized that decreasing glutamate release from dmPFC terminals in the BLA might attenuate withdrawal-related anxiety. To demonstrate the feasibility of the approach using chemogenetics, we coinjected ChR2- and G_i_-DREADD-expressing viruses into the dmPFC and, 6 weeks later, examined the effects of 10 μm CNO on oEPSC paired-pulse ratios recorded from BLA principal neurons *in situ*. CNO significantly decreased the glutamate paired-pulse ratio in both AIR animals (baseline: −0.26 ± 0.06; CNO: −0.05 ± 0.08, *N* = 6; paired *t* test, *t*_(5)_ = 3.70, *p* < 0.05; Fig. 4*A1*,*A2*) and CIE/WD animals (baseline, −0.62 ± 0.02; CNO, −0.38 ± 0.04, *N* = 6; paired *t* test, *t*_(5)_ = 6.35, *p* < 0.01; Fig. 4*B1*,*B2*). Importantly, CNO had no effect on glutamate paired-pulse ratios in recordings from animals lacking the G_i_-DREADDs (“ChR2 only”; baseline: −0.54 ± 0.04; CNO: −0.56 ± 0.05; *N* = 11; paired *t* test, baseline vs CNO: *t*_(10)_ = 0.98, *p* = 0.346; Fig. 4*C*). The percentage of inhibition of the first-response oEPSC amplitude by this concentration CNO was similar between AIR (68.3 ± 14.1% inhibition, *N* = 6) and CIE/WD (59.4 ± 12.8%, *N* = 6) animals (unpaired *t* test, *t*_(10)_ = 0.468, *p* = 0.65; Fig. 4*D*). These data demonstrate that the G_i_-DREADDs expressed on dmPFC terminals in the BLA can inhibit synaptic responses independent of the CIE exposure and can reduce the release probability in a way that was similar to those of other G_i_-coupled receptors.

### Bath application of CNO also decreases glutamate release from G_q_-DREADD-expressing vmPFC terminals in the BLA in air-exposed control animals

Because CIE decreases glutamate release from vmPFC terminals in the BLA, we hypothesized that G_q_-DREADDs could be used to increase glutamate release at these synapses. Although not statistically significant (unpaired *t* test, *t*_(15)_ = 1.727, *p* = 0.105), light-evoked paired-pulse ratios recorded from G_q_-expressing vmPFC–BLA synapses in air-exposed animals and in the absence of CNO (PPR: −0.78 ± 0.05, *N* = 4) appeared to be qualitatively different from those previously recorded from ChR2-only-expressing vmPFC–BLA synapses (PPR: −0.54 ± 0.04, *N* = 11; data from Fig. 4*C*). Surprisingly, bath application of CNO (10 μm) significantly decreased the amplitude of the oEPSCs recorded from G_q_-DREADD-expressing terminals by 36.78 ± 4.806% (paired *t* test: baseline: 79.6 ± 6.1 pA; vs CNO 51.0 ± 6.7 pA, *N* = 5; *t*_(4)_ = 10.24, *p* = 0.0005). Although the mechanism responsible for G_q_-DREADD inhibition of oEPSCs is not clear, these findings clearly suggest that local activation of these receptors in vmPFC-BLA terminals could not be used to facilitate glutamate release following chronic ethanol exposure.

### Chemogenetic inhibition of glutamate release from dmPFC terminals in the BLA decreases anxiety-like behavior during withdrawal

**Figure 5.**
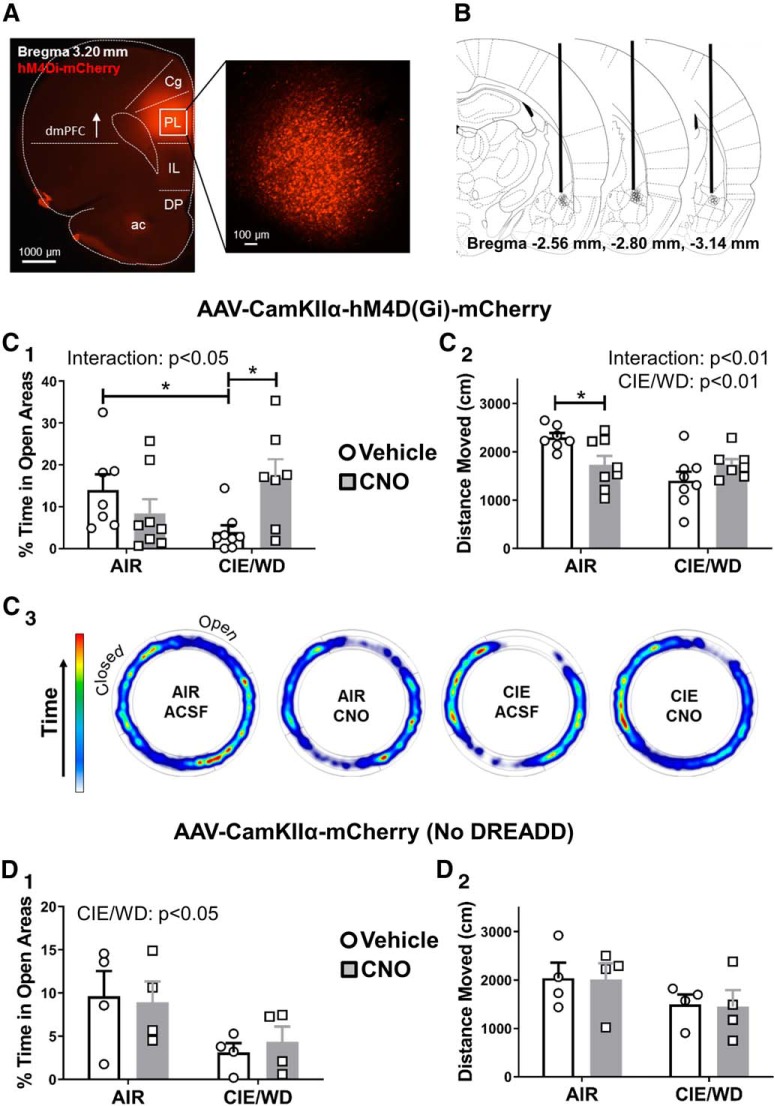
CNO-mediated inhibition of dmPFC-BLA terminals expressing the G_i_-DREADD attenuates withdrawal-induced anxiety-like behavior. ***A***, Representative fluorescent images of the PL-targeted injection site for the G_i_-coupled DREADDS (mCherry reporter). Inset, Right, Magnified view within the PL. ac, Anterior commissure. ***B***, Schematic depicting approximate the location of guide cannula tips (black circles). Anterior/posterior position relative to bregma is indicated at the bottom of each image (coronal slice diagrams are taken from Paxinos and Watson, 2005). ***C***, CNO application to dmPFC-BLA terminals reduces withdrawal-related anxiety-like behavior. ***C_1_***, Percentage of time spent in the open areas of the elevated zero maze for AIR (*N* = 15) and CIE/WD (*N* = 15) animals expressing G_i_-DREADDs that received microinjections of either aCSF (*N* = 7-8/group) or CNO (*N* = 7-8/group; 300 μm). Significant interaction between AIR/CIE and vehicle/CNO (see text; two-way ANOVA) along with significant differences between AIR ACSF vs CIE/WD aCSF and between CIE/WD aCSF vs CIE/WD CNO (Bonferroni post-tests, *p < 0.05). No significant differences between AIR aCSF and AIR CNO or between AIR aCSF and CIE/WD CNO. ***C_2_***, Significant interaction and the main effect of exposure condition (AIR vs CIE/WD) for the total distance moved in centimeters on the EZM (see text; two-way ANOVA). CNO significantly impacted locomotor behavior in air-exposed controls (Bonferroni’s post-test, *p < 0.05). ***C_3_***, Representative heat plots of the amount of time spent in open vs closed areas of the EZM depicting averaged group data. ***D***, The effects of CNO microinjection into the BLA require the expression of the G_i_-DREADD in dmPFC terminals. ***D_1_***, The main effect of exposure condition (AIR vs CIE/WD) on the percentage of time spent in the open areas of the EZM in animals without the DREADD construct (*N* = 4/group). ***D_2_***, No differences observed between any of the conditions for total distances moved. Figure originally published in Paxinos and Watson, 2005. Copyright Elsevier 2005.

Since activation of G_i_-coupled DREADDs with CNO decreases glutamate release from dmPFC terminals in the BLA, we examined the behavioral consequences of silencing this circuit during ethanol withdrawal. We microinjected the hM4Di-DREADD into the dmPFC (Fig. 5*A*) and implanted chronic cannulae into the BLA to deliver either vehicle (aCSF) or CNO directly to the dmPFC terminals. All of the cannulations were localized between −2.56 and −3.14 mm from bregma as revealed through the *post hoc* analysis (Fig. 5*B*). One of the air-exposed, aCSF-treated animals expressed significantly greater locomotor activation by the elevated zero maze (total distance traveled; *p* < 0.05, Grubb’s test) compared with the rest of this treatment group and was removed from the study. Similar to previous findings (Morales et al., 2018), withdrawal from 7 d of CIE increased anxiety-like behavior measured by the percentage of time spent in the open areas on the elevated zero maze (AIR treated − aCSF microinjected: 14.0 ± 3.7%, *N* = 7; CIE/WD − aCSF: 4.0 ± 1.6%, *N* = 8; EZM; Fig. 5*C1*). Importantly, microinjection of CNO (300 μm) into the BLA largely reversed the effects of CIE/WD and increased the amount of time that ethanol-exposed animals spent in the open areas of the EZM (CIE/WD–CNO: 17.0 ± 4.4%, *N* = 7; Fig. 5*C1*,*C3*). Two-way analysis of these data revealed a significant interaction (*F*_(1,26)_ = 7.68, *p* < 0.05) between the exposure (air vs CIE/WD) and treatment (vehicle vs CNO) factors. A planned *post hoc* analysis using Bonferroni’s post-test demonstrated a significant withdrawal effect (AIR vs CIE) only for animals in the aCSF treatment group (*t* = 2.564, *p* < 0.05), but not for animals treated with CNO (*t* = 1.737, *p* > 0.05). Bonferroni’s comparisons of treatment effect (aCSF vs CNO) showed a significant effect of CNO in the CIE/WD animals (*t* = 2.647, *p* < 0.05), but no significant effect of CNO in AIR-exposed control animals (AIR CNO: 8.4 ± 3.4%, *N* = 8; *t* = 1.17, *p* > 0.05). We used total distance moved during the assay as a proxy measure for locomotor activity (Fig. 5*C2*). A two-way ANOVA revealed a significant interaction (exposure × treatment: *F*_(1,26)_ = 8.46, *p* < 0.01) and a main effect of exposure group (AIR vs CIE/WD: *F*_(1,26)_ = 8.24, *p* < 0.001) but no significant main effect of CNO microinjection (*F*_(1,26)_ = 0.56, *p* = 0.460). While the preferential locomotor suppression by CNO in the air-exposed animals complicates interpretation of the anxiety-like behavior (see Discussion), these data together suggest that G_i_-DREADD inhibition of dmPFC terminals in the BLA can likewise attenuate withdrawal-related increases in anxiety-like behaviors.

### CNO has no effect in rats injected with the control virus lacking the G_i_-coupled DREADD

To examine whether CNO-mediated behavioral effects were specific to G_i_-DREADD expression in dmPFC-BLA terminals, we generated a separate cohort of animals that expressed a control virus lacking the G_i_-DREADD receptor (mCherry only) and again microinjected CNO into the BLA following either air or chronic ethanol exposure. A two-way ANOVA of the percentage of time spent in open area (Fig. 5*D1*) revealed a main effect of exposure (AIR vs CIE/WD: *F*_(1,12)_ = 6.627, *p* < 0.05), with no main effect of CNO treatment (*F*_(1,12)_ = 0.016, *p* = 0.903) and no interaction (exposure × treatment: *F*_(1,12)_ = 0.197, *p* = 0.665). Percentage time spent in open areas for animals with control virus is as follows: AIR aCSF: 9.6 ± 2.9%, *N* = 4; AIR CNO: 8.9 ± 2.4%, *N* = 4; CIE/WD aCSF: 3.1 ± 1.1%, *N* = 4; CIE/WD CNO: 4.3 ± 1.8%, *N* = 4. A two-way ANOVA of total distance traveled (Fig. 5*D2*) revealed no main effects or interactions, as follows: exposure condition (*F*_(1,12)_ = 3.22, *p* = 0.098); treatment group (*F*_(1,12)_ = 0.01578, *p* = 0.902); and interaction (*F*_(1,12)_ = 0.001529, *p* = 0.970; Fig. 5*G*). Total distance, in millimeters, is as follows: AIR aCSF: 2036 ± 321 cm, *N* = 4; AIR CNO: 2010 ± 335 cm, *N* = 4; CIE//WD aCSF: 1498 ± 205 cm, *N* = 4; and CIE/WD CNO: 1447 ± 345 cm, *N* = 4.

### Withdrawal from CIE increases the E/I ratio in the dmPFC–BLA pathway

**Figure 6.**
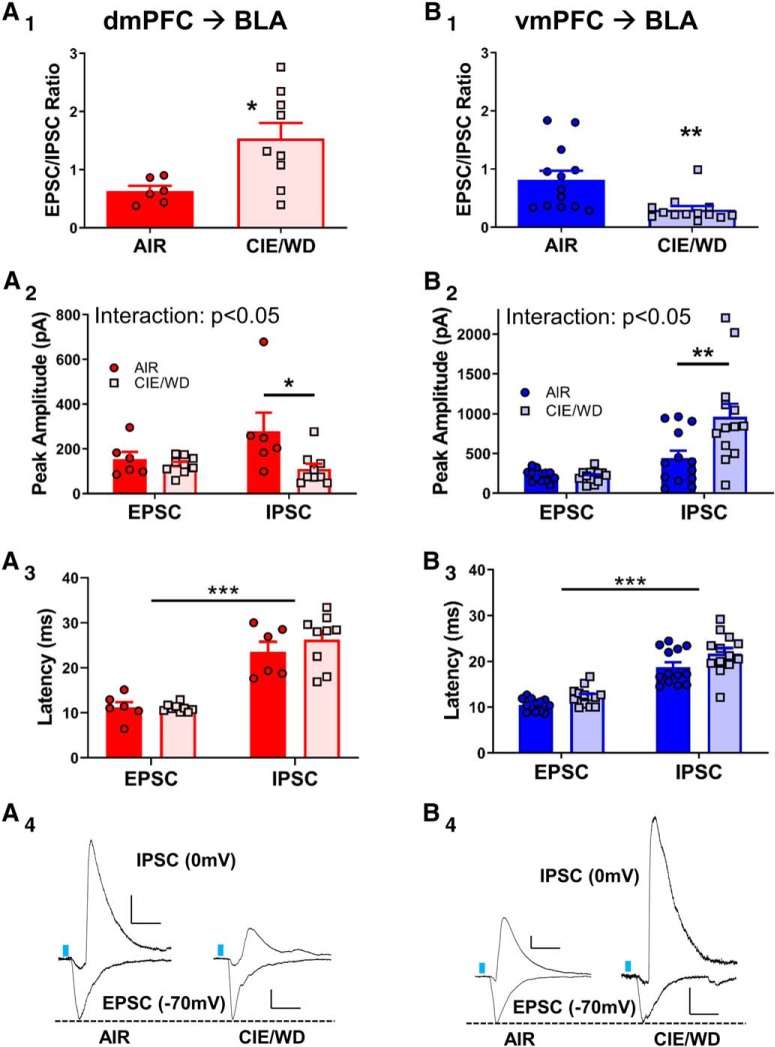
Withdrawal from CIE produces opposing alterations to the excitation/inhibition balance in the dmPFC and vmPFC BLA pathways. ***A_1_***, Relative to AIR controls (*N* = 6), CIE/WD (*N* = 9) significantly increased the light-evoked E/I ratio recorded from dmPFC-BLA terminals. *p < 0.05, unpaired *t* test. ***A_2_***, CIE/WD significantly decreased peak IPSC amplitudes relative to AIR controls in responses recorded from dmPFC–BLA synapses (see text; *p < 0.05, Bonferroni post-test following two-way ANOVA, significant interaction). ***A_3_***, Response onset latency for EPSCs and IPSCs recorded from dmPFC–BLA inputs. ISPCs had significantly longer latencies than EPSCs regardless of exposure condition (***p < 0.001, main effect of response type, two-way ANOVA). ***A_4_***, Representative traces recorded from dmPFC-BLA terminals in the two treatment groups. IPSCs recorded at 0 mV and EPSCs recorded at −70 mV. ***B_1_***, Light-evoked E/I ratios recorded from vmPFC-BLA terminals. Note the significant decrease (**p < 0.01, unpaired *t* test) in the E/I ratio in CIE/WD (*N* = 13) vs AIR (*N* = 13). ***B_2_***, Peak amplitudes of EPSCs and IPSCs recorded from vmPFC–BLA synapses. Significant interaction between treatment group and response type (see text; two-way ANOVA). Note the significant increase in the IPSC amplitude in CIE/WD neurons compared with AIR controls (***p < 0.001, Bonferroni’s multiple-comparisons post-test). ***B_3_***, Response onset latency for EPSCs and IPSCs recorded from vmPFC–BLA inputs. ISPCs had significantly longer latencies than EPSCs regardless of exposure condition (see text; two-way ANOVA, ***p < 0.001, main effect of response type). ***B_4_***, Representative traces recorded from vmPFC-BLA terminals. EPSC amplitudes were normalized across treatments to emphasize changes to the IPSC amplitude. Calibration: *x* = 40 ms, *y* = 50 pA. Dashed lines denote normalized oEPSC amplitudes across the treatment groups to emphasize the treatment effects on the IPSCs.

CNO modulation of behavior in the air-treated control animals was an unexpected observation. To gain a better understanding of potential circuit-mediated contributions to this effect (Fig. 5*C*), we examined the balance between excitatory and inhibitory transmission evoked by light stimulation of dmPFC terminals in the BLA. In the presence of the NMDA receptor antagonist APV (50 μm), we recorded optically evoked glutamatergic EPSCs at a holding potential of −70 mV followed by GABAergic IPSCs at 0 mV in the same cell. Since we have already shown that withdrawal from chronic ethanol increased glutamate transmission at these synapses (Fig. 2*C2*), we adjusted the laser intensity to produce equivalent EPSCs between treatment groups and measured the resulting IPSCs from that same stimulation intensity. We found that withdrawal from 7 d of CIE significantly increases the EPSC/IPSC ratio (Fig. 6*A1*,*A4*; AIR: 0.633 ± 0.088, *N* = 6; CIE/WD: 1.536 ± 0.266, *N* = 9; unpaired *t* test, AIR vs CIE/WD: *t*_(13)_ = 2.677, *p* < 0.05). As expected, peak amplitudes of the oEPSCs were not significantly different under these stimulation conditions (Fig. 6*A2*; AIR: 154.2 ± 32.3 pA, *N* = 6; CIE/WD: 123.8 ± 12.5 pA, *N* = 10; unpaired *t* test, AIR vs CIE/WD: *t*_(13)_ = 0.832, *p* = 0.420). However, IPSCs were significantly smaller in the CIE/WD group (130.4 ± 29.97 pA, *N* = 10) compared with the AIR group (278.1 ± 83.4, *N* = 6; unpaired *t* test, AIR vs CIE/WD: *t*_(13)_ = 2.305, *p* < 0.05). This suggests that the treatment-dependent effects on the E/I ratio is driven by a decrease in the average peak amplitude of IPSCs recorded from CIE/WD animals. For the dmPFC, the latency of response onset, measured in milliseconds poststimulation, was significantly longer for light-evoked IPSCs (AIR: 23.3 ± 1.9 ms, *N* = 7; CIE/WD: 25.9 ± 1.6 ms, *N* = 10) relative to the EPSCs measured in the same neurons (AIR: 11.3 ± 0.7 ms; CIE/WD: 11.0 ± 0.3 ms) regardless of exposure condition (Fig. 6*A3*; two-way ANOVA: interaction: *F*_(1,30)_ = 1.293, *p* = 0.265; treatment: *F*_(1,30)_ = 0.828, *p* = 0.370; response type: *F*_(1,30)_ = 107.1, *p* < 0.001). These data, along with the TTX/4-AP data (Fig. 1*C2*), suggests that the oEPSCs are monosynaptic, while the IPSCs may arise from a local, multisynaptic circuit. Supporting this, in a representative group of cells from both treatment groups these IPSCs were inhibited by both picrotoxin (100 μm) and DNQX (20 μm). In contrast, electrically evoked IPSCs in the BLA are sensitive to picrotoxin alone (Lindemeyer et al., 2014). These findings suggest that the GABAergic responses recorded from optical activation of dmPFC terminals in the BLA likely represent local feedback-type circuits. These circuits provide a potential mechanism for increased anxiety-like behaviors following CNO microinjection into air-treated animals expressing the G_i_-DREADD in dmPFC-BLA terminals.

### Withdrawal from CIE decreases the E/I ratio in the vmPFC–BLA pathway

In contrast to the dmPFC–BLA projection, recent work within the IL–BLA projection suggests that these latter synapses may directly engage feedforward inhibition (Cho et al., 2013). We therefore performed the same experiments for vmPFC-BLA terminals. In contrast to the dmPFC–BLA pathway, withdrawal from 7 d of CIE significantly decreased the EPSC/IPSC ratio in the vmPFC–BLA pathway (Fig. 6*B1*). An unpaired *t* test was used to compare the E/I ratios from AIR (0.82 ± 0.15, *N* = 13) and CIE/WD (0.30 ± 0.06) animals (*t*_(24)_ = 3.152, *p* < 0.01). Again, oEPSC peak amplitudes were not significantly different between air-exposed animals (221.7 ± 20.1 pA) and CIE/WD animals (Fig. 6*B2*; 212.2 ± 20.9 pA; unpaired *t* test, *t*_(24)_ = 0.329, *p* = 0.745). Conversely, IPSC amplitudes were significantly increased in CIE/WD neurons (958.6 ± 164.3 pA) relative to AIR controls (442.5 ± 93.2 pA; unpaired *t* test, *t*_(24)_ = 2.73, *p* < 0.05). Similar to the dmPFC pathway, the latencies of IPSCs (AIR: 18.8 ± 1.1 ms, *N* = 13; CIE/WD: 21.7 ± 1.2 ms, *N* = 13) were significantly longer than those of EPSCs (AIR: 10.5 ± 0.4 ms; CIE/WD: 12.4 ± 0.6 ms) recorded from optically stimulating the vmPFC pathway (Fig. 6*B3*). A two-way ANOVA revealed main effects of response type (EPSC vs IPSC: *F*_(1,48)_ = 103.2, *p* < 0.0001) and of exposure (CIE/WD latencies > AIR latencies by ∼2 ms; *F*_(1,48)_ = 7.646, *p* < 0.01), but no interaction (response × exposure: *F*_(1,48)_ = 0.378, *p* = 0.388). Similar to the dmPFC responses, EPSCs evoked from vmPFC terminals were sensitive only to DNQX while IPSCs were attenuated by the application of either picrotoxin (GABAergic) or DNQX (circuit dependent). While these data do not rule out unique contributions of potential feedforward GABAergic circuits within the vmPFC–BLA pathway, they nonetheless suggest substantial contributions by local, feedback circuits as well.

## Discussion

The present study sought to examine the effects of chronic ethanol exposure and withdrawal on glutamate release probability at inputs arising from two subdivisions of the medial prefrontal cortex to the basolateral amygdala. We used a combination of optogenetics and chemogenetics to document the neurophysiological and behavioral alterations of dmPFC and vmPFC terminals in the BLA. The major findings of the study are that withdrawal from chronic ethanol exposure increases optogenetically stimulated glutamate release from dmPFC-BLA terminals but decreases the glutamate release from vmPFC terminals. Additionally, we demonstrate that chemogenetic inhibition of dmPFC terminals in the BLA reduces withdrawal-induced anxiety-like behavior. And finally, we show that local feedback-like GABAergic circuits make significant contributions to optically gated responses from both dmPFC and vmPFC terminals. Together, these data provide the first characterization of how chronic ethanol exposure and withdrawal changes mPFC–BLA circuitry and suggests a novel role for this circuit in regulating ethanol withdrawal-associated anxiety.

Through precise stereotaxic microinjection, we targeted the dmPFC and vmPFC. To prevent unintended viral spread between the dmPFC and vmPFC regions, we placed the tip of our injectors in the top third of the PL and the bottom third of the IL. This allowed for maximal separation of dmPFC and vmPFC but caused spread of the virus into the anterior cingulate cortex (Cg) and dorsal peduncular cortex (DP) in some cases. Recently, the anterior cingulate cortex and its input to the basolateral amygdala has been implicated in mediating innate freezing response to predator odors (Jhang et al., 2018), which is similar to the role the PL plays in learned fears (Corcoran and Quirk, 2007). Although the projection from the dorsal peduncular cortex to the BLA has not been directly characterized, the DP is thought to play a role similar to the IL and is often included when examining conditioned responses such as fear and drug seeking (Peters et al., 2009). Hence, we refer broadly to the dmPFC and the vmPFC. These are common subdivisions based on cytoarchitecture, functional criteria, and connectivity with other brain regions (Heidbreder and Groenewegen, 2003). Notably, homologies among the human, primate, and rodent prefrontal cortices exist, which allow for cross-species comparisons (Laubach et al., 2018). The pattern of terminal expression in the BLA was similar between dmPFC- and vmPFC-injected animals such that only cells in the anterior aspect of the basolateral nucleus were innervated, and this was consistent along the rostral–caudal axis. We also noted that glutamate release probabilities (PPR ratios) were similar along the anteroposterior axis of the BLA. It is also noteworthy that, relative to the BLA, the lateral and central nuclei of the amygdala were largely devoid of fluorescence arising from both dmPFC and vmPFC. These findings are consistent with previous reports (Arruda-Carvalho et al., 2017).

The use of TTX and 4-AP to demonstrate monosynaptic circuits is imperative when using optogenetics and electrophysiology. Without these pharmacological manipulations, it is difficult to distinguish light-evoked responses originating within transfected terminals from optical activation of local networks. In the present study, the application of TTX, which inhibits action potential firing, completely blocked oEPSCs evoked from both the dmPFC and vmPFC. And, the addition of 4-AP and the subsequent blockade of voltage-gated potassium channels, which theoretically prolong ChR2-mediated terminal depolarization, are sufficient to “rescue” terminals from TTX inhibition and trigger glutamate release (Fig. 1). These findings are entirely consistent with previous reports that mPFC–BLA projections are monosynaptic (Cho et al., 2013; Little and Carter, 2013; Arruda-Carvalho and Clem, 2014; McGarry and Carter, 2017; Kiritoshi and Neugebauer, 2018).

Previous studies have characterized the presynaptic and postsynaptic adaptations that occur following withdrawal from chronic ethanol exposure. Using electrical stimulation, for example, electrophysiology recordings have shown that withdrawal from CIE exposure increases the glutamate release probability at medial stria terminalis synapses (Christian et al., 2013; Morales et al., 2018) and that this facilitation requires just 3 d of intermittent ethanol exposure (Morales et al., 2018). Accordingly, we wanted to investigate whether these findings were representative for inputs from specific brain areas. Projections from the mPFC to the BLA arrive via the medial stria terminalis (Sesack et al., 1989), suggesting that these synapses were appropriate candidates to undergo presynaptic alterations following withdrawal from chronic ethanol exposure. Surprisingly, we found that withdrawal from 3 d of CIE did not alter the glutamate release probability from either dmPFC or vmPFC synapses (Fig. 2), while a withdrawal from a 7 d exposure was sufficient. This apparent differential sensitivity to the number of ethanol exposures in different studies may be driven by a number of factors, as follows: (1) dmPFC and vmPFC inputs require longer CIE exposures relative to the entire population of stria terminalis inputs activated by electrical stimulation; (2) optically evoked release, in general, requires longer CIE exposures than electrically evoked release; or (3) the age of the animals during the CIE exposure changes the relative sensitivity of stria terminalis inputs to chronic ethanol exposure. We cannot exclude the possibility that dmPFC/vmPFC inputs are more resistant to CIE relative to other stria inputs, given current technical limitations. However, with respect to the CIE sensitivity of optically evoked versus electrically evoked release, baseline paired-pulse ratios from optical stimulation of dmPFC and vmPFC inputs in air-exposed animals are much lower (release is higher) than is typically seen with electrically evoked release from the stria terminalis population of synapses. Despite the higher basal release probability with optical stimulation, dmPFC and vmPFC inputs are significantly different from each other at baseline and yet express opposite responses to CIE. This suggests that the basal release probability at any synapse, regardless of the stimulation approach, may not dictate its relative CIE sensitivity. Notably, in the study using electrical stimuli of the stria (Christian et al., 2013; Morales et al., 2018), animals were exposed to ethanol during early adolescence [postnatal day 39 (P39) to P46)], whereas animals used in the present study were adults at the time of ethanol exposure (P70+). This was necessitated by the use of optogenetics and chemogenetics, which require a prolonged period for transgene expression and transport of channelrhodopsin/DREADD receptor to the terminal fields. There are numerous studies highlighting the vulnerability of adolescents to ethanol relative to adults (for review, see Crews et al., 2016). Therefore, our data are consistent with the hypothesis that adult-like animals are more resilient and thus require a longer ethanol exposure to induce similar synaptic alterations seen in adolescent animals.

We found that withdrawal from 7 d of CIE produces opposite effects on glutamate release probability from dmPFC and vmPFC projections to the BLA. Notably, the dmPFC and vmPFC play distinct roles in both fear conditioning and drug-seeking behaviors: the dmPFC drives the expression of fear and drug seeking; and, the vmPFC suppresses these behaviors following extinction learning (for review, see Peters et al., 2009). Fear learning also leads to strengthening of PL, but not IL, excitatory synapses onto BLA principal neurons (Arruda-Carvalho and Clem, 2014). Additionally, the relative strengths of synaptic inhibition and excitation within the IL–BLA pathway, but not the PL–BLA pathway, are increased in an arthritic pain model (Kiritoshi and Neugebauer, 2018). A large body of evidence in both rats and humans suggests that both the cellular mechanisms and the circuits underlying fear and anxiety behaviors extensively overlap (for review, see Davis et al., 2010; Tovote et al., 2015). Thus, increased glutamate release from dmPFC terminals may promote fear learning, anxiety, and drug seeking, whereas the decreased glutamate release from vmPFC terminals diminishes the ability of these projections to act in opposition. Our findings therefore show that glutamate release from two mPFC–BLA circuits, which serve independent and, in some ways, opposing functions, are differentially regulated by a dependence-like ethanol exposure.

Notably, glutamate release probabilities, measured using paired-pulse ratios, were significantly different between dmPFC (low release)-BLA and vmPFC (high release)-BLA terminals in ethanol-naive control animals (Fig. 2). To our knowledge, no previous studies have directly compared glutamate release from these two pathways in rats. However, one study in mice reported equivalent recruitment of excitation and feedforward inhibition onto BLA principal neurons by these pathways (Arruda-Carvalho and Clem, 2014). Regardless, differences in “basal” glutamate transmission suggests that, under “normal” circumstances, the higher release from vmPFC inputs would dominate top–down control over the BLA and potentially help prevent maladaptive behavior responses. Conversely, pathologic states like alcohol dependence/withdrawal reverse the relative release probabilities at these inputs and accentuate top–down control by dmPFC inputs, which could ultimately drive maladaptive behaviors. It is also worth noting that alterations in synchronized activity between mPFC and amygdala have been reported in humans with alcoholism (Müller-Oehring et al., 2015), although the behavioral implications of these changes were not directly assessed. Baseline and “pathological” differences in release probability may be related to the size of pool of readily releasable presynaptic vesicles or alterations in calcium dynamics within the terminals (Fioravante and Regehr, 2011). Given that calcium plays an integral role in neurotransmitter release and directly regulates short-term changes in release probability (for review, see Neher and Sakaba, 2008; Südhof, 2012; Catterall et al., 2013), we directly examined the extent to which extracellular calcium played in determining glutamate release probability from dmPFC-BLA and vmPFC-BLA terminals. We found that reducing extracellular calcium levels affected the release probability of ethanol-naive vmPFC synapses but not dmPFC inputs, suggesting that differences in calcium dynamics within the terminals may underlie the differences in synaptic strength and the differential effect of chronic ethanol within these two circuits.

GABAergic transmission in the BLA tightly regulates the activity of principal neurons via both feedforward and feedback inhibition. Chronic ethanol exposure selectively decreases GABA release from feedforward neurons without altering the release from local interneurons (Diaz et al., 2011). In the present study, we examined the excitatory/inhibitory ratios expressed at dmPFC and vmPFC synapses in the BLA (Fig. 6) in both air-exposed control animals and those in withdrawal. Similar to our findings with glutamate, we found that the E/I ratios were differentially altered in these two pathways such that the E/I ratio was increased at dmPFC–BLA synapses and decreased at vmPFC–BLA synapses. Likewise, while the approaches used here do not allow us to differentiate between feedforward and feedback GABAergic responses, our findings do suggest that feedback-like IPSCs contribute substantially to the E/I ratios measured with optical stimulation of both dmPFC and vmPFC terminals. This is evidenced by significantly longer onset latencies for IPSC relative to the EPSCs. Regardless, these E/I data suggest that CIE/WD may reduce the recruitment of intrinsic GABAergic circuits from dmPFC terminals while recruiting a more extensive intrinsic GABA circuit activated by vmPFC inputs. This interpretation strictly requires that similar numbers of dmPFC and vmPFC afferents are activated during a given optical stimulus; and, it is technically impossible to determine this directly with the approaches used here. Nonetheless, the terminal field fluorescent intensities arising from these brain regions within the BLA appear to be quite similar (Fig. 1).

The BLA has been implicated in a wide range of behaviors (for review, see Janak and Tye, 2015); and, increased glutamate signaling within the BLA is specifically associated with increased expression of emotional behaviors like fear and anxiety (for review, see McCool et al., 2010). For example, microinjection of the glutamate receptor antagonist DNQX into the BLA attenuates anxiety-like behavior expressed during withdrawal (Läck et al., 2007). In line with this, our current findings show that microinjection of CNO into the BLA diminishes withdrawal-related anxiety-like behavior (Fig. 5), presumably through activation of G_i_-coupled DREADDs expressed in dmPFC terminals. While CNO microinjection specifically increased open-area times in the CIE/WD animals, the control animals in this study spent a modest amount of time in the open areas relative to previous reports using other assays (Morales et al., 2018). While it is possible that the elevated zero maze is particularly provocative relative to the elevated plus maze or the light/dark transition test, recent work with general anesthetics (Landin et al., 2019) showed that adolescent exposure to general anesthetics can enhance the avoidance of novel objects and decrease social interactions, an effect that persists at 3–7 weeks after the exposure. The modest open area times in the current study may therefore reflect behavioral interactions between surgical anesthesia during adolescence and CIE exposures several weeks later. Regardless, CIE/WD animals spent significantly less time in the open areas compared with the air-exposed controls. Our EZM data thus directly demonstrate that dmPFC projections to the BLA play an important role in regulating anxiety-like behavior. Although projections from the BLA to the mPFC can modulate anxiety-related behaviors (Felix-Ortiz et al., 2016), the behavioral contributions of reciprocal mPFC projections to the BLA has not been as extensively explored.

We should note that CNO microinjection appeared to differentially suppress locomotor behavior (distance moved) in the air-exposed, DREADD-expressing control animals. While this limits the interpretation of EZM data to some extent, locomotor suppression was not apparent following CNO microinjection into the “no DREADD” controls for this study, suggesting that it is directly related to CNO actions on the dmPFC terminals near the guide cannula. In addition to CNO actions in the BLA, it is possible that the diffusion of CNO into neighboring structures could have played some role. However, the relative fluorescent intensity of YFP (Fig. 1*C*) suggests that the majority of dmPFC inputs into this brain region are largely focused within the basolateral nucleus, consistent with existing literature (Cassell and Wright, 1986; Sesack et al., 1989; McDonald et al., 1996; Likhtik et al., 2005). The modulation of BLA principle neuron activity via local manipulation of dmPFC projections can influence a number of downstream brain regions. For example, BLA principal neurons project directly to the central amygdala. Given that CIE exposure appeared to alter the activity of local GABAergic circuitry (Fig. 6), our behavioral findings in the Air-DREADD animals suggest that interactions between this intrinsic BLA inhibitory circuitry and the dmPFC inputs may have driven responses to CNO microinjection.

Given the functional role of the vmPFC and that CIE and withdrawal decreases glutamate release from vmPFC terminals in the BLA, we hypothesized that artificially increasing glutamate release from these inputs would attenuate anxiety-like behavior during withdrawal. In support of this, electrical stimulation of the IL increases the time spent and entries in the open arms of an elevated-plus maze, suggesting a key role in downregulating anxiety-like behavior (Shimizu et al., 2018). This parallels the well established role that this region plays in extinction learning. Before testing our hypothesis *in vivo*, we wanted to examine the effects of G_q_-coupled DREADD activation in these vmPFC–BLA projections. To our surprise, CNO activation of the G_q_-DREADD resulted in a decrease of glutamate release rather than the predicted increase. Interestingly, we also noted that paired-pulse ratios recorded from vmPFC terminals expressing ChR2 and G_q_-DREADDS qualitatively had greater CNO-independent release. One possible explanation is that G_q_-DREADD overexpression in vmPFC synaptic terminals results in some level of constitutive (agonist-independent) receptor activity. Although G_i/o_-coupled autoreceptors and heteroreceptors are mainly known to inhibit evoked transmitter release, there is also evidence that the activation of G_q_-coupled receptors can cause both synaptic inhibition and facilitation (Betke et al., 2012). Since the G_q_-DREADDs did not increase glutamate release *in vitro*, we did not attempt to use this approach *in vivo*.

In conclusion, our results indicate a novel role of discrete mPFC inputs to the BLA beyond that of conditioned fear. We demonstrate that the dmPFC–BLA pathway is involved in regulating anxiety and that chemogenetic inhibition of these projections during alcohol withdrawal can decrease withdrawal-induced anxiety-like behavior. These data suggest that withdrawal from chronic ethanol exposure may selectively strengthen dmPFC synapses while weakening vmPFC synapses in the BLA. Strategies focused on reversing these alterations may offer targets for treatment development. Future studies will examine the molecular mechanisms underlying the ethanol-induced synaptic plasticity within these two mPFC–BLA circuits.

## Synthesis

Reviewing Editor: Lorna Role, NINDS Decisions are customarily a result of the Reviewing Editor and the peer reviewers coming together and discussing their recommendations until a consensus is reached. When revisions are invited, a fact-based synthesis statement explaining their decision and outlining what is needed to prepare a revision will be listed below. The following reviewer(s) agreed to reveal their identity: Danny Winder, Lawrence Chandler.

Overall, the reviewers saw this as a very interesting (potentially news worthy) set of studies exploring alterations in glutamate inputs from two different PFC sites into the BLA in response to chronic alcohol exposure, and the potential contributions of these alterations to rodent behavior. Circuit specificity of inputs was achieved using both chemo and optogenetic viral approaches combined with both electrophysiological and behavioral measurements, with the intriguing finding that CIE appears to have opposing actions on dmPFC versus vmPFC inputs in the BLA.

Although this is, on balance, a well written and well executed set of studies that provide the first look at the important mPFC-BLA connection in ethanol withdrawal, both reviewers had significant issues with the behavioral studies presented in figure 5.

As such the authors are asked to revise the presentation of the behavioral data and to modify all aspects of the discussion to carefully reflect the limitations of their data.

These required revisions are detailed below under “major issues”.

In addition, other comments and some minor corrections are also listed below (under additional and minor issues) that should be attended to prior to resubmission.

Major:

1. Significant concerns relate to the behavioral data:

a. In figure 5, there appears to be a strong trend for a decrease in open area time in the air controls after CNO in Fig 5C1, consistent with the significant result in Fig 5C2. The altered distance phenotypes make interpretation slightly more complicated, though Fig 5D1 and Fig 5D2 help in this regard.

The reviewers are concerned that another possible interpretation of the data is that the % time phenotype is driven by a differential CNO locomotor suppression in the Air vs CIE mice. Although it would be best to see data from a second behavioral task used to corroborate these findings, the reviewers will settle for a detailed discussion of the data limitations.

b. As shown in Figure 5C1 and 5D1, that rats only spend 10-15% of their time in the open areas. This suggests that environmental conditions of the EZM were fairly aversive and may have biased observations and introduced a floor effect. Again, the best resolution of these issues would be to repeat the experiments with more optimized test conditions (e.g. reduced lighting) such that the rats spend more time in the open (e.g. 30-40%). Please address this issue in the revised manuscript.

c. The authors noted in text that an advantage of the EZM over the EPM is that it does not have the “ambiguous” center position. The reviewers do not entirely agree with this comment. Looking at Figure 5C3, the rats spend much of their time at the border for light and dark crossing which could also be argued as being ambiguous. The reviewers recommend deleting the comment from the methods section.

d. The authors are asked to use best practices of presenting the data as scatter plots rather than bar graphs to better assess the data.

e. the authors should comment on the relative spread of their manipulations between the BLA and CeA, given the clear role of the CeA in anxiety-related behaviors.

Additional:

i. please include the graphs of the data (bars with mean SD and/or scatter plots) in figure 4A1.

ii. Regarding the extracellular calcium experiments - the reviewers express concern that 1 mM calcium should be referred to as “reduced” extracellular calcium (rather than “low”)

iii. Can the authors comment on why they did not observe the changes after 3 days of CIE? Do they have any new data (or published data) looking a dependence withdrawal scores after 3 versus 7 days of CIE?

iv. Please indicate how many times/which days blood was obtained for CIE. Did this include all animals or just a subset?

Minor:

i. what was the volume of CNO that was injected?

ii. Top of the second page of the introduction: Suggest rephrasing “a leading neurobiological...”.

iii. Page 13, line 8: Delete “a”

iv. Page 13, last line: insert “is” between “It” and “well”

v. In the discussion, the authors seem to imply that they had directly compared synaptic strength in these cases, but since I/O curves were not generated, the authors should restrict their discussion to the release probability argument specifically.

vi. The authors should be careful with the discussion at the top of page 24, as this argument presumes the similar numbers of afferents from the different mPFC regions would be activated.

## Author Response

We are grateful for the reviewer's helpful comments and have extensively revised the manuscript to address their concerns. Reviewers noted that our studies were very interesting (potentially news worthy) and were well-written and -executed. Below we address the major and minor concerns individually noting modifications to the text where necessary.

Major:

1. Significant concerns relate to the behavioral data:

a. In figure 5, there appears to be a strong trend for a decrease in open area time in the air controls after CNO in Fig 5C1, consistent with the significant result in Fig 5C2. The altered distance phenotypes make interpretation slightly more complicated, though Fig 5D1 and Fig 5D2 help in this regard.

The reviewers are concerned that another possible interpretation of the data is that the % time phenotype is driven by a differential CNO locomotor suppression in the Air vs CIE mice.

We agree with respect to the open area time in Fig 5C1 - these data prompted our exploration of the EPSC/IPSC data in Fig 6 which offers a potential neurophysiological mechanism. During re-plotting of the data to include the requested scatter plots (see below), we noted that one animal in the Air-Vehicle group was a significant outlier with respect to the rest of the population for locomotor-related behaviors (distance moved, p<0.05 with a Grubb's). This outlier was removed from the results (explicitly noted in the Results section of the manuscript); but this did not alter the statistical results for either ‘distance moved’ (revised Fig. 5C2) or the open area time (revised Fig. C1). Regardless, given the absence of any CNO-related locomotor effects in the “No DREADD” controls (revised Fig. 5D2), we agree the data supports the reviewers' interpretation that CNO caused differential locomotor suppression in the Air-exposed controls. The discussion section (8th paragraph) has been modified to reflect this interpretation. We have also provided a more detailed discussion of the limitations of these data.

b. As shown in Figure 5C1 and 5D1, that rats only spend 10-15% of their time in the open areas. This suggests that environmental conditions of the EZM were fairly aversive and may have biased observations and introduced a floor effect. Again, the best resolution of these issues would be to repeat the experiments with more optimized test conditions (e.g. reduced lighting) such that the rats spend more time in the open (e.g. 30-40%). Please address this issue in the revised manuscript.

We agree that floor/ceiling effects are always a limitation when measuring anxiety-like behaviors in ‘open area avoidance’ assays; and, we have always utilized pilot studies to ascertain the appropriate conditions to minimize this possibility. Based on pilot studies with surgerized, CIE-exposed animals, the current study employed a 7.5W incandescent bulb during the elevated zero-maze assay (this is now explicitly noted in the Methods section). This level of illumination represents the lower-limit possible with our equipment. Although LED-based illumination could theoretically provide lower illumination, our initial experience with these types of systems suggests that the light they produce tends to be more focused and is more difficult to standardize across all portions of the apparatus compared to incandescent lighting. Notably, CIE/WD-vehicle animals spent significantly less time in the open areas of the EZM compared to Air-vehicle controls (revised Fig. 5C1 and D1). This strongly suggests that any ‘floor’ effect was not sufficient enough to undermine interpretation of the open area data.

With respect to the modest amounts of time animals spent in the open areas, we agree with the reviewers that 10-15% is less than desirable. We have consistently observed (since 2007) that surgerized, CIE-exposed animals express greater anxiety-like behavior than either ‘surgerized’-only or ‘CIE exposed’-only animals regardless of the assay employed. Recent work (Landin et al 2019 Neuropharmacol. 150: 153) showed that adolescent exposure (P28) to isoflurane enhanced avoidance of a novel object and reduced social interactions even when measured 3-6.5 weeks after the exposure. We therefore believe the modest open area times in the current study reflect behavioral interactions between the surgical procedures employing isoflurane (two exposures in this case) and the CIE treatment. We now highlight these potential interactions in the Discussion section (8th paragraph) to explain greater anxiety-like behavior in the Air animals in this study relative to published work that did not employ exposures to general anesthetics.

c. The authors noted in text that an advantage of the EZM over the EPM is that it does not have the “ambiguous” center position. The reviewers do not entirely agree with this comment. Looking at Figure 5C3, the rats spend much of their time at the border for light and dark crossing which could also be argued as being ambiguous. The reviewers recommend deleting the comment from the methods section.

This comment has been deleted.

d. The authors are asked to use best practices of presenting the data as scatter plots rather than bar graphs to better assess the data.

Scatter plots have been added to all the relevant figure panels.

e. the authors should comment on the relative spread of their manipulations between the BLA and CeA, given the clear role of the CeA in anxiety-related behaviors.

We acknowledge that potential spread of CNO to the neighboring CeA is possible and now explicitly state this in the discussion (9th paragraph, a new paragraph). It should be noted that the relative fluorescent intensity in Fig. 1C suggests that the BLA, not CeA, receives the vast majority of inputs from the dmPFC. This is consistent with a large body of literature using classic tract-tracing methods and in vivo electrophysiology (citations in text).

Additional:

i. please include the graphs of the data (bars with mean SD and/or scatter plots) in figure 4A1.

These are now included. We assumed that we should include them for Fig. 4 B1 and C as well.

ii. Regarding the extracellular calcium experiments - the reviewers express concern that 1 mM calcium should be referred to as “reduced” extracellular calcium (rather than “low”)

The figures and text have been revised to reflect this request

iii. Can the authors comment on why they did not observe the changes after 3 days of CIE? Do they have any new data (or published data) looking a dependence withdrawal scores after 3 versus 7 days of CIE?

We now provide additional discussion in the 4th paragraph related to the different exposure durations in different studies. With respect to dependence withdrawal scores, published work shows that withdrawal from 3d & 7d CIE both decrease open arm time in the elevated plus maze; unpublished findings show that 7d, but not 3d, decreases light-side time in the light-dark box. This suggests that withdrawal from increasing durations of CIE produces increases in withdrawal-related anxiety-like behavior that is dependent upon the assay being employed. Beginning with the fourth paragraph of the discussion, the original submission explicitly contrasted the current findings with previous work showing that 3 days was sufficient to produce increases in electrically-evoked glutamate release from stria terminalis. This discussion highlighted differences in the age of the animals in the current study (P70+) compared to previous work using electrical stimulation (P39-46). We have expanded the discussion to now include the following additional possibilities: 1) dmPFC and vmPFC require longer exposures compared to inputs from other brain regions represented within the stria terminalis population of synapses; and, 2) optically evoked release requires longer exposures than electrically evoked release.

With respect to the unique nature of dm/vmPFC inputs, unpublished work shows that polymodal sensory thalamic inputs to the lateral amygdala (P70+ animals) require longer CIE exposures to express increased optically-evoked release. So, our limited experience with these approaches suggest that distinct inputs may not be unique with respect to the CIE duration required to produce presynaptic changes in glutamate release. With respect to optically-evoked versus electrically-evoked release, baseline paired-pulse ratios with optical stimulation from dmPFC and vmPFC inputs in air-exposed and naïve animals are much lower (release is higher) than is typically seen with electrically-evoked release from the population of synapses within the stria inputs. It is therefore possible that robust optically-evoked release could alter the duration CIE exposure required to measure changes in glutamate release. However, dmPFC and vmPFC inputs have unique release probabilities at baseline (both higher than electrically-evoke release) and express completely opposite responses to CIE. This suggests that the basal release probability at any synapse may not dictate the magnitude (or direction) of its sensitivity to CIE. While we cannot directly exclude any of these possible mechanisms (age, unique nature of the input, or unique contributions by optically-evoked release), we provide this detailed response to support the hypothesis that the age of the animals is likely to make a significant contribution to the differences between the CIE durations required in the current work versus those reported for electrical stimulation.

iv. Please indicate how many times/which days blood was obtained for CIE. Did this include all animals or just a subset?

This is now in the “Methods” section.

Minor:

i. what was the volume of CNO that was injected?

This was found in the “Elevated Zero Maze” subheading of the “Materials and Methods” section of the original submission. 0.5μL over a 1min period, injectors left in place for an additional minute. Animals placed on the EZM 5min later.

ii. Top of the second page of the introduction: Suggest rephrasing “a leading neurobiological...”.

This phrase has been deleted

iii. Page 13, line 8: Delete “a”

Deleted

iv. Page 13, last line: insert “is” between “It” and “well”

Edited

v. In the discussion, the authors seem to imply that they had directly compared synaptic strength in these cases, but since I/O curves were not generated, the authors should restrict their discussion to the release probability argument specifically.

We have attempted to remove any indications of a comparison between dmPFC and vmPFC synaptic strength.

vi. The authors should be careful with the discussion at the top of page 24, as this argument presumes the similar numbers of afferents from the different mPFC regions would be activated.

We agree with the reviewer's excellent point. We have modified the discussion in the 7th paragraph to reflect this limitation in the interpretation of the E/I ratio data.
